# Acute ingestion of dietary nitrate increases muscle blood flow via local vasodilation during handgrip exercise in young adults

**DOI:** 10.14814/phy2.13572

**Published:** 2018-01-22

**Authors:** Jennifer C. Richards, Matthew L. Racine, Christopher M. Hearon, Megan Kunkel, Gary J. Luckasen, Dennis G. Larson, Jason D. Allen, Frank A. Dinenno

**Affiliations:** ^1^ Human Cardiovascular Physiology Laboratory Department of Health and Exercise Science Center for Cardiovascular Research Colorado State University Fort Collins Colorado USA; ^2^ Medical Center of the Rockies Foundation Poudre Valley Health System Loveland Colorado USA; ^3^ Department of Kinesiology Curry School of Education University of Virginia Charlottesville Virginia USA; ^4^ Duke Molecular Physiology Institute Duke University Durham North Carolina USA

**Keywords:** Dietary nitrate, exercise hyperemia, oxygen consumption

## Abstract

Dietary nitrate (${\text{NO}}_{3}^{-}$) is converted to nitrite (${\text{NO}}_{2}^{-}$) and can be further reduced to the vasodilator nitric oxide (NO) amid a low O_2_ environment. Accordingly, dietary ${\text{NO}}_{3}^{-}$ increases hind limb blood flow in rats during treadmill exercise; however, the evidence of such an effect in humans is unclear. We tested the hypothesis that acute dietary ${\text{NO}}_{3}^{-}$ (via beetroot [BR] juice) increases forearm blood flow (FBF) via local vasodilation during handgrip exercise in young adults (*n* = 11; 25 ± 2 years). FBF (Doppler ultrasound) and blood pressure (Finapres) were measured at rest and during graded handgrip exercise at 5%, 15%, and 25% maximal voluntary contraction (MVC) lasting 4 min each. At the highest workload (25% MVC), systemic hypoxia (80% SaO_2_) was induced and exercise continued for three additional minutes. Subjects ingested concentrated BR (12.6 mmol nitrate (*n* = 5) or 16.8 mmol nitrate (*n* = 6) and repeated the exercise bout either 2 (12.6 mmol) or 3 h (16.8 mmol) postconsumption. Compared to control, BR significantly increased FBF at 15% MVC (184 ± 15 vs. 164 ± 15 mL/min), 25% MVC (323 ± 27 vs. 286 ± 28 mL/min), and 25% + hypoxia (373 ± 39 vs. 343 ± 32 mL/min) and this was due to increases in vascular conductance (i.e., vasodilation). The effect of BR on hemodynamics was not different between the two doses of BR ingested. Forearm VO_2_ was also elevated during exercise at 15% and 25% MVC. We conclude that acute increases in circulating ${\text{NO}}_{3}^{-}$ and ${\text{NO}}_{2}^{-}$ via BR increases muscle blood flow during moderate‐ to high‐intensity handgrip exercise via local vasodilation. These findings may have important implications for aging and diseased populations that demonstrate impaired muscle perfusion and exercise intolerance.

## Introduction

During exercise, blood flow increases to contracting skeletal muscles primarily via local vasodilation, and this increase in blood flow and thus oxygen delivery occurs in proportion to the metabolic demand of the tissue (Anrep and von Saalfeld 1935; Bockman 1983; Bockman et al. 1980; Mohrman and Regal 1988). Mechanical effects of muscle contraction on the resistance vessels, K^+^ released during muscle depolarization, as well as a host of other candidate vasodilator substances such as ATP, prostaglandins, and nitric oxide (NO), all appear capable of participating in the local vasodilation observed during exercise (Clifford and Hellsten 2004). Regarding the latter, NO can be produced enzymatically by different NO synthase isoforms located on endothelial cells (eNOS) as well as via neuronal NOS (nNOS) on skeletal muscle cells, and NO synthesis increases with elevations in intracellular calcium levels that can occur in response to muscle contractions (Pearson et al. 2015; Pye et al. 2007; Stamler and Meissner 2001). Furthermore, following ingestion, dietary nitrate (${\text{NO}}_{3}^{-}$) is actively taken up into the salivary glands and once in the presence of commensal bacteria within the oral cavity (Lundberg et al. 2008), is reduced to nitrite (${\text{NO}}_{2}^{-}$), an important storage pool that can be further reduced to NO in the presence of reductase enzymes located on myoglobin, hemoglobin and also within eNOS and skeletal muscle (Lundberg et al. 2008; Piknova et al. 2016, 2015). The enzymatic activity of the reductase enzymes is elevated during conditions of lowered PO_2_ and pH such as during hypoxia and exercise (Crawford et al. 2006; Lundberg et al. 2008; Zweier et al. 1995).

Recently, there has been great interest in understanding the metabolic and vascular effects of dietary ${\text{NO}}_{3}^{-}$, and a number of studies have reported that ${\text{NO}}_{3}^{-}$ consumption can enhance exercise tolerance and performance by reducing the O_2_ cost of exercise (Bailey et al. 2010, 2009; Lansley et al. 2011a,b; Larsen et al. 2010), likely due to enhanced mitochondrial efficiency and/or an improved P/O ratio (Larsen et al. 2011). Early studies in humans indicate that direct ${\text{NO}}_{2}^{-}$ infusion into the local arterial circulation of the forearm increases blood flow at rest and during exercise via reduction in NO, and this effect is enhanced during systemic hypoxia (Cosby et al. 2003; Dejam et al. 2007; Maher et al. 2008). Consistent with these findings, Ferguson et al. recently demonstrated that 5 days of dietary ${\text{NO}}_{3}^{-}$ supplementation (via beetroot juice [BR]) significantly increased skeletal muscle blood flow via local vasodilation during exercise in healthy rats, an effect that was most pronounced in fast twitch muscle fibers (Ferguson et al. 2013). In contrast, the two studies to date in young healthy humans (Casey et al. 2015; Kim et al. 2015) using acute ${\text{NO}}_{3}^{-}$ supplementation via BR have failed to replicate these findings in rats. Although it is unclear why these studies did not demonstrate an increase in exercising muscle blood flow, issues related to the magnitude of increase in plasma ${\text{NO}}_{2}^{-}$, as well as exercise intensity and/or exercise duration may have contributed. Furthermore, it is possible that the reduction in metabolic cost with ${\text{NO}}_{3}^{-}$ supplementation resulted in less metabolic vasodilation and masked any potential increase in skeletal muscle blood flow due to the augmented ${\text{NO}}_{2}^{-}$ to NO conversion.

Our present understanding regarding the effect of ${\text{NO}}_{3}^{-}$ supplementation on metabolic cost during exercise is derived from studies utilizing walking, running, and cycling with measures of pulmonary VO_2_ (Bailey et al. 2015; Jones 2014; Lansley et al. 2011a,b; Vanhatalo et al. 2010; Wylie et al. 2013a). To the best of our knowledge, no studies have measured exercising limb blood flow and venous blood gasses to calculate active skeletal muscle oxygen consumption. Following dietary ${\text{NO}}_{3}^{-}$ consumption, if there is a reduction in the O_2_ cost of exercise, it is feasible that skeletal muscle blood flow to the working muscles may be attenuated in an effort to match O_2_ delivery to muscle O_2_ demand. Conversely, similar to the findings in rats (Ferguson et al. 2013), enhanced NO production with dietary ${\text{NO}}_{3}^{-}$ may facilitate greater peripheral vasodilation in the exercising skeletal muscle bed, leading to greater skeletal muscle blood flow despite an attenuated O_2_ cost.

Accordingly, the aim of the present study was to determine whether acute dietary ${\text{NO}}_{3}^{-}$ supplementation with BR increases forearm blood flow (FBF) via local vasodilation and lowers the O_2_ cost of exercise during graded handgrip exercise in both normoxic and hypoxic conditions. We hypothesized that compared with control conditions, consumption of BR would significantly increase muscle blood flow and vascular conductance and lower forearm VO_2_ during graded handgrip exercise in young healthy humans.

## Methods

### Subjects

With Colorado State University Institutional Review Board approval and following written informed consent, a total of 18 young healthy subjects participated in the present study (Table 1). All participants were nonsmokers, nonobese, normotensive, and not taking any medications including over the counter supplements. Additionally, all participants refrained from using mouthwash for 24 h prior to study participation as mouthwash has been observed to impede the reduction of ${\text{NO}}_{3}^{-}$ to ${\text{NO}}_{2}^{-}$ (Govoni et al. 2008). Female subjects were studied during the early follicular phase of the menstrual cycle or placebo phase of contraceptive use. Studies were performed after a 12‐h fast with the subjects in the supine position. The experimental arm of the subject was slightly elevated above heart level to minimize any potential influence of the muscle pump on forearm hemodynamics. All studies were performed according to the Declaration of Helsinki. Additionally, subjects were blinded to whether they received BR or placebo.

**Table 1:** Subject characteristics

Beet juice group	
Male:Female	6:5
Age (years)	25 ± 2
BMI (kg/m^2^)	23 ± 1
Maximal voluntary contraction (kg)	35 ± 4
Forearm volume (mL)	823 ± 106
Placebo group	
Male:Female	5:2
Age (years)	24 ± 1
BMI (kg/m^2^)	23 ± 1
Maximal voluntary contraction (kg)	42 ± 3
Forearm volume (mL)	1077 ± 39

Initially, five participants ingested 210 mL (12.6 mmol) of dietary nitrate (Beet It Sport Shot, James White Drinks, Suffolk, UK) and our preliminary analysis revealed a modest increase (~12–15%) in exercising FBF 2 h after consumption. Previous studies in animals reported ~40% increase in hind limb blood flow during treadmill exercise in rats (Ferguson et al. 2013), so in an effort to ensure participants had an optimal dose for us to observe any change in FBF, we increased the dose (280 mL/16.8 mmol) and the duration (3 h) that we waited (Wylie et al. 2013a) prior to starting the BR trial in the subsequent participants (*n* = 6). Seven additional subjects underwent the same experimental protocol but ingested placebo shots (210 or 280 mL) free of dietary nitrate (James White Drinks) and served as a time control group. Both BR and placebo subjects were blinded to which shots they received.

### Venous catheterization and blood gas measurements

An 18‐gauge catheter (3.8 cm) was inserted in retrograde fashion into a deep antecubital vein of the experimental arm for venous blood samples draining skeletal muscle (Crecelius et al. 2011; Richards et al. 2015). Saline was continuously infused through this catheter at a rate of approximately 3 mL/min for the duration of the study to keep it patent (Crecelius et al. 2011). Venous blood samples were immediately analyzed at rest and during exercise with a clinical blood gas analyzer (Siemens Rapid Point 405 Automatic Blood Gas System, Los Angeles, CA) for partial pressures of venous oxygen and carbon dioxide (*P*O_2_ and *P*CO_2_), venous oxygen content (ctO_2_), pH, and oxygen saturation (*S*O_2_).

### Forearm blood flow, vascular conductance, and oxygen consumption

A 12‐MHz linear‐array ultrasound probe (Vivid 7; General Electric, Milwaukee, WI) was used to determine brachial artery mean blood velocity (MBV) and brachial artery diameter. For blood velocity measurements, the probe insonation angle was maintained at <60 degrees and the frequency used was 5 MHz. The Doppler shift frequency spectrum was analyzed via a Multigon 500V TCD (Multigon Industries, Mt Vernon, NY) spectral analyzer from which mean velocity was determined as a weighted mean of the spectrum of Doppler shift frequencies. Brachial artery diameter measurements were made in duplex mode at end‐diastole at rest and between contractions (in triplicate) during steady‐state conditions. Forearm blood flow was calculated as:

FBF = MBV × π(brachial artery diameter/2)^2^ × 60, where the FBF is in mL/min, the MBV is in cm/s, the brachial diameter is in cm, and 60 was used to convert from mL/s to mL/min. A fan was directed toward the experimental arm to minimize the potential contribution of skin blood flow to forearm hemodynamics.

As an index of vascular tone, forearm vascular conductance (FVC) was calculated as: (FBF/Mean Arterial Pressure) × 100 and expressed as (mL/min/100 mmHg) (Crecelius et al. 2010).

Forearm VO_2_ was calculated as: FBF × (arterial – venous O_2_ content). Arterial oxygen content was calculated as: CaO_2_ = (Hb × 1.36 × SaO_2_) + PaO_2_ × 0.003, where SaO_2_ was measured via pulse oximetry (see below) and PaO_2_ at rest and during 80% SaO_2_ was estimated based on previously published arterial blood gas data in young adults in our laboratory (Richards et al. 2017). Importantly, several studies including those from our laboratory have shown that arterial oxygen content does not change during forearm (handgrip) exercise in humans (Casey et al. 2010; Crecelius et al. 2011).

### Heart rate and mean arterial pressure

Heart rate (HR) was monitored with a 3‐lead ECG. Mean arterial pressure (MAP) was measured by placing a finger pressure cuff around the middle phalanx of the middle finger on the nonexperimental arm, which remained at heart level (supine position) throughout the measurement (Finometer; Finapres Medical Systems BV, Amsterdam, The Netherlands). Resting arterial blood pressure was measured over the brachial artery following 30 min of supine rest and just prior to each exercise trial (Cardiocap 5; Datex Ohmeda, Louisville, CO), and resting Finometer MAP was corrected for differences between the two readings (Kirby et al. 2005).

### Handgrip exercise

Maximal voluntary contraction was determined for each subject as the average of at least three maximal squeezes of a handgrip dynamometer (Stoelting, Chicago, IL) that were within 3% of each other. Subjects lifted a weight corresponding to their % MVC, 4–5 cm over a pulley using both audio and visual cues to ensure correct timing of contraction (1 sec: 0.5 sec concentric, 0.5 sec eccentric) and relaxation (2 sec) (Dinenno and Joyner 2003, 2004).

### Systemic hypoxia

During the highest workload (25% MVC), hypoxia was elicited utilizing utilized a self‐regulating partial rebreathe system (Banzett et al. 2000) which allows for constant alveolar fresh air ventilation independent of changes in minute ventilation and enables end‐tidal CO_2_ (EtCO_2_) to be clamped (Banzett et al. 2000; Crecelius et al. 2011). Oxygen (O_2_) levels were titrated down by mixing nitrogen with air in a medical gas blender to attain steady arterial O_2_ saturations (SaO_2_) of 80% as assessed by pulse oximetry (SpO_2_) of the earlobe. Nasal breathing was prevented through the use of a nose clip while subjects breathed through a scuba mouthpiece. An anesthesia monitor was used to monitor gas concentrations at the level of the mouthpiece (Cardiocap; Datex‐Ohmeda) as well as to monitor heart rate (3‐lead ECG). Additionally, ventilation was measured with a pneumotachograph (model VMM‐2a; Interface Associates, Laguna Niguel, CA).

### Plasma measures of nitrate (${\text{NO}}_{3}^{-}$) and nitrite (${\text{NO}}_{2}^{-}$)

Prior to the start of each trial (control and BR) plasma ${\text{NO}}_{3}^{-}$ and ${\text{NO}}_{2}^{-}$ samples were collected whereby 3–4 mL of blood was drawn from a vein in the antecubital region and centrifuged at 5000 *g* for 6 min at 4°C. Plasma was removed and immediately frozen via liquid nitrogen and stored (−80°C) for later analysis (Ferguson et al. 2013). Due to hemolysis of baseline samples, only samples from eight of the 11 participants in the BR group were analyzed for plasma ${\text{NO}}_{3}^{-}$ and ${\text{NO}}_{2}^{-}$ concentrations via chemiluminescence with an Ionic/Sievers NO analyzer (NOA 280i; Sievers Instruments, Boulder, CO). All measurements of plasma ${\text{NO}}_{3}^{-}$ and ${\text{NO}}_{2}^{-}$ were analyzed within 15 min of thawing. Potassium iodide in acetic acid was used to reduce ${\text{NO}}_{2}^{-}$ to NO because it is unable to reduce ${\text{NO}}_{3}^{-}$, and is therefore more specific to ${\text{NO}}_{2}^{-}$. This reductant possesses the ability to reduce NO_2_^−^ to NO but is incapable of reducing higher oxides of nitrogen (i.e., NO_3_^−^), thus increasing the specificity for NO_2_^−^. Plasma ${\text{NO}}_{3}^{-}$ concentrations were determined using the reductant vanadium chloride in hydrochloric acid at 95°C, which reduces all nitrogen oxides with an oxidation state of +2 or higher including ${\text{NO}}_{3}^{-}$ (μmol/L) and NO_2_^−^ (nmol/L) (Allen et al. 2009).

### Experimental protocols

**Figure 1:**
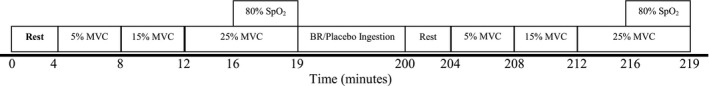
Study Timeline. Following venous catheter insertion and 30 min of quiet rest, participants performed graded handgrip exercise (5%, 15%, 25% maximal voluntary contraction [MVC]) for 4 min at each intensity. During 25% MVC, participants continued performing handgrip exercise for an additional 3 min while they underwent systemic hypoxia (80% SpO_2_). The blood flow response to graded exercise and hypoxic exercise was assesses in control conditions and following ingestion of 210 or 280 mL of concentrated BR (12.6 mmol nitrate (*n* = 5) or 16.8 mmol nitrate (*n* = 6). Participants performed their second bout (beetroot [BR] juice) of exercise either 2 h (12.6 mmol) or 3 h (16.8 mmol) postconsumption. On a separate day and with separate individuals (*n* = 7), the experimental protocol was repeated with nitrate‐free concentrated BR juice. Venous blood was obtained for ${\text{NO}}_{3}^{-}$ and ${\text{NO}}_{2}^{-}$ at rest prior to the onset of each exercise trial, and for blood gasses at rest and the end of each exercise intensity during normoxia and hypoxia.

Figure 1 provides a timeline for the study day (BR and placebo). Each exercise trial consisted of 2 min of resting baseline followed by continuous incremental handgrip exercise: 4 min each at 5%, 15%, and 25% MVC. Following 2 min or attainment of steady‐state FBF at the third workload (25% MVC), systemic isocapnic hypoxia (80%) began and subjects continued to perform handgrip exercise. For three additional minutes or until steady‐state FBF values were observed, 80% SpO_2_ was maintained. These workloads are equivalent to ~15%, 40%, and 70% of maximum work rate (Richards et al. 2014). Venous blood samples were obtained at rest for plasma ${\text{NO}}_{3}^{-}$ and ${\text{NO}}_{2}^{-}$ and blood gas measures, and at the end of each exercise, intensity for blood gas measures. Subjects then ingested BR or placebo, and the protocol was repeated 2–3 h postconsumption (Wylie et al. 2013a).

### Statistics

Data are presented as mean ± SEM. Within each protocol, differences between trials were determined via two‐way repeated measures analysis of variance (ANOVA). Post hoc comparisons were made with the Holm–Sidak test. Significance was set at *P *<* *0.05.

## Results

### Subjects

Subject characteristics are presented in Table 1. Eleven subjects received BR and seven received placebo shots. Systemic and forearm hemodynamics (Table 2), venous blood gasses (Table 3), and ventilation, ETCO_2_, and SpO_2_% (Table 4), for all subjects during control and BR or placebo conditions are presented in tabular form.

**Table 2:** Hemodynamics at rest and during exercise in BR and Placebo Trials

	HR (bpm)	MAP (mmHg)	Brachial artery diameter (mm)	FBF (mL/min)	FVC (mL/min/mmHg)	VO_2_ (mL/min)
Control trial						
Rest	60 ± 3	87.2 ± 2.0	3.8 ± 0.1	29.2 ± 4.7	33.3 ± 5.0	2.2 ± 0.2
5% MVC	61 ± 3	90.4 ± 2.2	3.8 ± 0.1	59.4 ± 4.7	65.0 ± 3.9	6.9 ± 0.6
15% MVC	65 ± 3	92.1 ± 2.6	3.9 ± 0.1	164.4 ± 15.1	199.6 ± 15.4	20.1 ± 2.3
25% MVC	70 ± 3	98.3 ± 3.0	4.0 ± 0.1	285.8 ± 28.5	288.6 ± 22.5	33.9 ± 4.4
25% MVC + hypoxia	83 ± 3	99.7 ± 2.7	4.1 ± 0.1	342.7 ± 31.9	341.1 ± 25.7	34.5 ± 5.4
Beet juice trial						
Rest	64 ± 3^a^	87.0 ± 2.3	3.8 ± 0.1	27.1 ± 2.8	31.2 ± 3.0	2.6 ± 0.2
5% MVC	66 ± 3^a^	87.9 ± 2.4	3.8 ± 0.1	63.5 ± 6.8	71.9 ± 6.7	7.6 ± 0.8
15% MVC	69 ± 3^a^	91.6 ± 3.0	3.9 ± 0.1	183.9 ± 14.9^a^	228.3 ± 17.6^a^	23.7 ± 2.9^a^
25% MVC	73 ± 3^a^	97.7 ± 2.9	4.0 ± 0.1	322.5 ± 27.1^a^	329.9 ± 24.3^a^	37.6 ± 4.7^a^
25% MVC + hypoxia	91 ± 5	100.8 ± 3.6	4.1 ± 0.1	372.9 ± 38.8^a^	369.9 ± 31.1^a^	37.8 ± 6.0
Control trial						
Rest	59 ± 3	90.7 ± 2.6	4.0 ± 0.2	30.4 ± 2.2	41.3 ± 7.2	2.3 ± 0.6
5% MVC	63 ± 4	93.4 ± 3.7	4.0 ± 0.2	75.0 ± 7.5	80.6 ± 7.5	8.4 ± 0.3
15% MVC	67 ± 4	95.4 ± 3.8	4.1 ± 0.2	197.6 ± 17.9	206.8 ± 14.7	26.2 ± 2.7
25% MVC	73 ± 4	99.0 ± 3.6	4.3 ± 0.1	338.7 ± 35.9	340.6 ± 29.1	43.8 ± 4.0
25% MVC + hypoxia	81 ± 4	100.6 ± 4.0	4.4 ± 0.2	417.9 ± 52.8	411.5 ± 38.1	54.8 ± 4.9
Placebo trial						
Rest	63 ± 5	92.0 ± 1.1	4.0 ± 0.2	29.5 ± 3.9	31.9 ± 3.9	3.4 ± 0.5
5% MVC	67 ± 5^a^	92.2 ± 2.3	4.0 ± 0.2	75.1 ± 11.0	81.3 ± 10.8	9.9 ± 1.2
15% MVC	69 ± 5	93.3 ± 2.1	4.1 ± 0.2	178.7 ± 23.4	192.7 ± 26.0	29.5 ± 3.9
25% MVC	75 ± 4	98.4 ± 2.1	4.2 ± 0.1	332.5 ± 32.2	338.5 ± 32.4	46.2 ± 4.3
25% MVC + hypoxia	82 ± 5	100.7 ± 2.8	4.4 ± 0.2	419.6 ± 38.2	416.6 ± 36.1	59.5 ± 5.5

a *P* < 0.05 versus control trial in respective condition.

**Table 3:** Venous blood gas variables

	pH	PCO_2_ (mmHg)	PO_2_ (mmHg)	SO_2_ (%)	p50 (mmHg)	CtO_2_ (mL/dL)
Control trial						
Rest	7.37 ± 0.01	47.9 ± 1.9	31.0 ± 2.5	55.0 ± 5.3	26.9 ± 0.6	10.7 ± 1.0
5% MVC	7.36 ± 0.01	50.2 ± 1.6	22.7 ± 1.2	36.5 ± 3.3	26.8 ± 0.4	7.3 ± 0.7
15% MVC	7.33 ± 0.02	55.2 ± 2.3	22.8 ± 0.6	35.6 ± 1.5	26.7 ± 0.5	7.1 ± 0.4
25% MVC	7.30 ± 0.02	59.7 ± 2.9	24.6 ± 1.0	37.4 ± 2.2	26.8 ± 0.6	7.5 ± 0.3
25% MVC + hypoxia	7.32 ± 0.02	55.6 ± 3.0	21.9 ± 1.6	31.6 ± 3.7	27.0 ± 0.5	6.8 ± 0.8
Beet juice trial						
Rest	7.36 ± 0.01^a^	46.2 ± 1.4	27.2 ± 1.6^a^	47.7 ± 4.1	26.7 ± 0.3	9.7 ± 0.8
5% MVC	7.36 ± 0.01	49.2 ± 2.2	22.1 ± 1.3	35.0 ± 3.3	26.9 ± 0.5	7.0 ± 0.7
15% MVC	7.32 ± 0.01	54.5 ± 2.2	22.5 ± 0.9	33.9 ± 1.9	26.7 ± 0.5	6.8 ± 0.3
25% MVC	7.29 ± 0.02	57.6 ± 3.4	25.6 ± 1.2	38.9 ± 2.5	26.8 ± 0.4	7.9 ± 0.4
25% MVC + hypoxia	7.31 ± 0.02^a^	53.2 ± 2.3	21.6 ± 1.2	30.7 ± 2.8	26.8 ± 0.5	6.7 ± 0.6
Control trial						
Rest	7.37 ± 0.01	43.6 ± 3.2	28.6 ± 2.7	48.3 ± 4.5	28.6 ± 1.7	10.4 ± 0.8
5% MVC	7.37 ± 0.01	50.2 ± 1.5	20.9 ± 0.7	35.3 ± 3.4	25.5 ± 0.7	7.6 ± 0.9
15% MVC	7.32 ± 0.01	57.5 ± 1.3	20.3 ± 0.8	29.8 ± 1.6	25.8 ± 0.6	6.4 ± 0.5
25% MVC	7.27 ± 0.01	63.4 ± 2.0	21.5 ± 0.9	30.0 ± 2.0	25.9 ± 0.3	6.5 ± 0.5
25% MVC + hypoxia	7.28 ± 0.01	63.0 ± 1.2	19.7 ± 0.9	24.3 ± 1.7	27.1 ± 0.1	5.2 ± 0.4
Placebo trial						
Rest	7.34 ± 0.01	49.0 ± 1.0	21.7 ± 1.3	33.6 ± 3.4	26.3 ± 0.2	7.4 ± 1.0
5% MVC	7.34 ± 0.01	54.0 ± 0.9	20.1 ± 0.3	28.0 ± 1.3	27.1 ± 0.8	5.9 ± 0.6
15% MVC	7.30 ± 0.02	59.6 ± 1.4	20.3 ± 1.0	28.5 ± 2.0	25.9 ± 0.3	6.2 ± 0.4
25% MVC	7.30 ± 0.01	63.8 ± 1.0	21.5 ± 0.8	29.0 ± 1.5	26.1 ± 0.5	6.3 ± 0.4
25% MVC + hypoxia	7.26 ± 0.01	64.6 ± 1.4	18.1 ± 1.7	22.6 ± 3.1	26.0 ± 1.5	4.8 ± 0.6

a *P* < 0.05 versus control condition in BR condition.

**Table 4:** Ventilation and pulse oximetry in BR and placebo trials

	Ve (L/min)	EtCO_2_ (mmHg)	SpO_2_ (%)
Control trial			
Rest	8.5 ± 0.7	36.3 ± 1.3	97.8 ± 0.2
5% MVC	8.4 ± 0.7	36.3 ± 0.9	97.5 ± 0.3
15% MVC	9.3 ± 0.5	37.3 ± 0.9	98.5 ± 0.7
25% MVC	11.3 ± 0.7	37.1 ± 0.9	98.3 ± 0.5
25% MVC + hypoxia	26.2 ± 0.2	35.8 ± 0.9	79.6 ± 0.7
Beet juice trial			
Rest	9.0 ± 0.6	36.9 ± 0.9	97.7 ± 0.3
5% MVC	9.9 ± 0.5^a^	36.7 ± 0.9	97.6 ± 0.4
15% MVC	10.2 ± 0.5^a^	36.7 ± 0.8	97.8 ± 0.5
25% MVC	12.6 ± 0.8^a^	37.1 ± 0.9	97.4 ± 0.4
25% MVC + hypoxia	31.2 ± 3.8^a^	36.7 ± 0.7	80.0 ± 0.6
Control trial			
Rest	9.5 ± 0.9	36.1 ± 1.8	97.8 ± 0.5
5% MVC	9.5 ± 0.8	37.1 ± 1.0	97.6 ± 0.9
15% MVC	10.3 ± 0.6	37.2 ± 0.9	97.5 ± 0.3
25% MVC	11.9 ± 0.7	37.1 ± 0.9	97.6 ± 0.3
25% MVC + hypoxia	31.1 ± 1.4	36.9 ± 0.7	79.8 ± 0.2
Placebo trial			
Rest	9.8 ± 0.5	35.3 ± 1.1	97.8 ± 0.5
5% MVC	11.4 ± 0.9	35.7 ± 1.0	97.5 ± 0.3
15% MVC	11.0 ± 0.5^a^	35.9 ± 1.0	97.3 ± 0.6
25% MVC	12.9 ± 1.1	35.9 ± 0.9	97.3 ± 0.7
25% MVC + hypoxia	35.7 ± 1.8^a^	36.5 ± 0.8	80.4 ± 0.9

a *P* < 0.05 versus control condition in respective trial.

### Plasma measures of nitrate and nitrite

**Figure 2:**
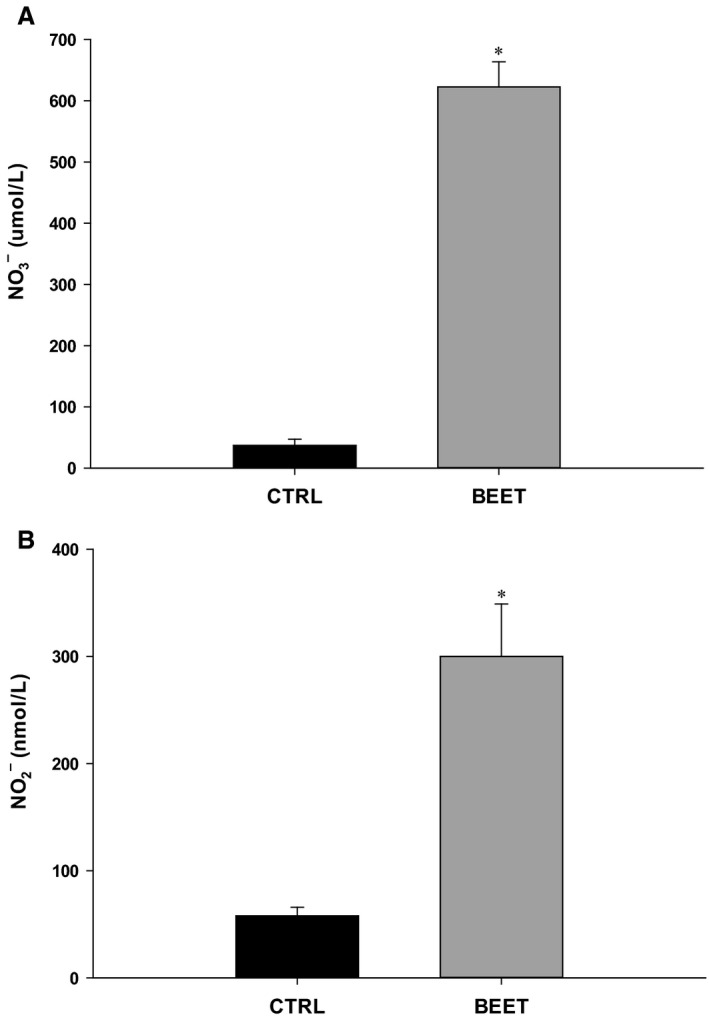
(A) Plasma Nitrate (${\text{NO}}_{3}^{-}$) and (B) Nitrite (${\text{NO}}_{2}^{-}$) concentrations in control trial and 2–3 h after ingestion of BR (BEET). *P < 0.05 versus control trial.

Resting venous plasma ${\text{NO}}_{3}^{-}$ and ${\text{NO}}_{2}^{-}$ concentrations were (37 ± 10 μmol/L and 58 ± 8 nmol/L) and both increased significantly 2–3 h after ingesting BR (622 ± 41 μmol/L and 301 ± 49 nmol/L, all *P* < 0.05) (*N* = 8, Fig. 2). There were no significant differences in the change in plasma ${\text{NO}}_{3}^{-}$ (609 ± 67 vs. 561 ± 56 μmol/L) between subjects that received 12.6 (*n* = 5) or 16.8 mmol (*n* = 3) of ${\text{NO}}_{3}^{-}$ via ingestion of BR, respectively. There was a greater increase in plasma ${\text{NO}}_{2}^{-}$ (340 ± 84 vs. 171 ± 35 nmol/L; *P* = 0.04) in the participants that received 16.8 mmol of ${\text{NO}}_{3}^{-}$, however, this greater rise in plasma ${\text{NO}}_{2}^{-}$ did not translate to a greater improvement in exercising muscle blood flow. We did not collect plasma samples for ${\text{NO}}_{3}^{-}$ or ${\text{NO}}_{2}^{-}$ from subjects who received the placebo shots since previous studies clearly indicate that there is no change in plasma concentrations of ${\text{NO}}_{3}^{-}$ and ${\text{NO}}_{2}^{-}$ following placebo (${\text{NO}}_{3}^{-}$ free) BR ingestion (Kim et al. 2015; Lansley et al. 2011b; Wylie et al. 2013b).

### Forearm blood flow and vascular conductance

**Figure 3:**
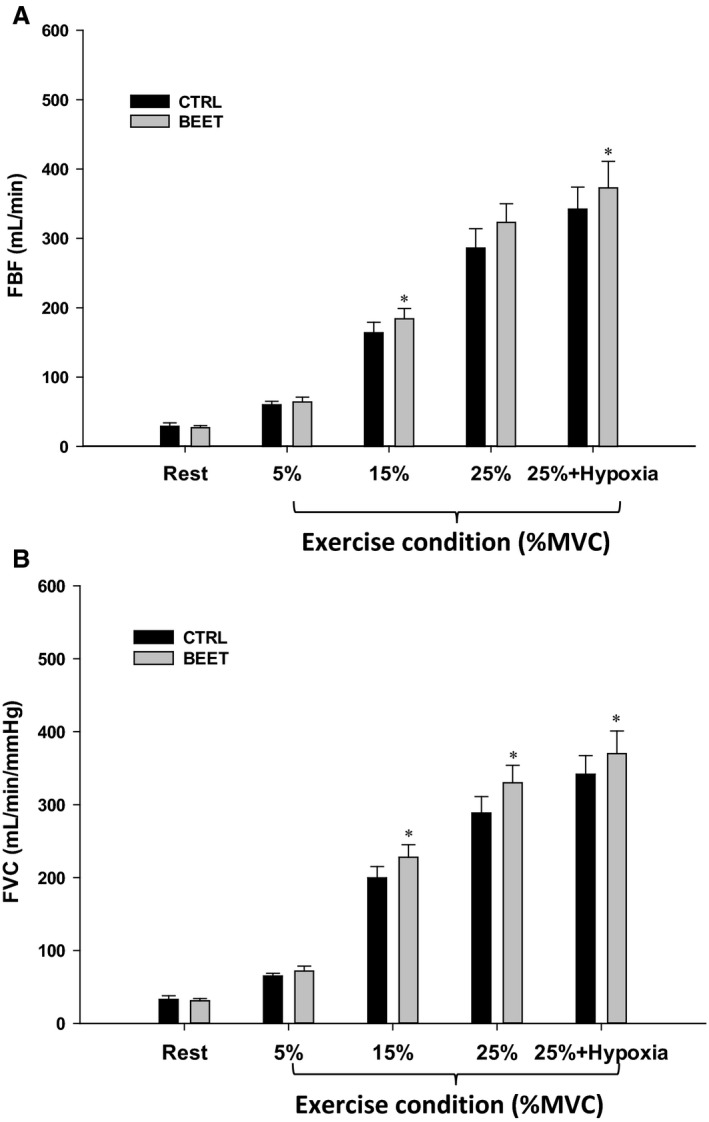
(A) Forearm blood flow and (B) vascular conductance during graded handgrip exercise and hypoxic exercise prior to and following ingestion of concentrated BR (BEET). *P < 0.05 versus control trial within respective condition (BEET or Placebo). % MVC, % of maximal voluntary contraction.

Resting FBF was not different between the two conditions (Control and BR; 29 ± 5 vs. 27 ± 3 mL/min; Table 2 and Fig. 3A). Following BR ingestion, FBF was not different during 5% MVC, but was significantly greater during 15% MVC (184 ± 15 vs. 164 ± 15 mL/min), 25% MVC (322 ± 27 vs. 286 ± 28 mL/min), and 25% MVC + hypoxia (373 ± 39 vs. 343 ± 32 vs. mL/min, all *P* < 0.05; Fig. 3A). There were no differences in brachial artery diameter at rest or during exercise within the two conditions (Control vs. BR; Table 2).

Similar to FBF, FVC was not different at rest (Control and BR) (33 ± 3 vs. 31 ± 3 mL/min/mmHg; Table 2 and Fig. 3B) and during 5% MVC, but was significantly increased following BR at 15% MVC (228 ± 17 vs. 199 ± 15 mL/min/mmHg), 25% MVC (330 ± 24 vs. 288 ± 22 mL/min/mmHg) and 25% MVC + hypoxia (369 ± 31 vs. 341 ± 25 mL/min/mmHg, all *P* < 0.05; Fig. 3B). In the placebo trial, there were no differences in FBF or FVC between the two conditions (Control vs. Placebo) at any time point (Table 2). Absolute values of FBF, FVC, and VO_2_ in these subjects (Placebo) were higher than subjects who received BR due to greater absolute workloads as MVC was ~20% greater (see Table 1).

### Forearm oxygen consumption

**Figure 4:**
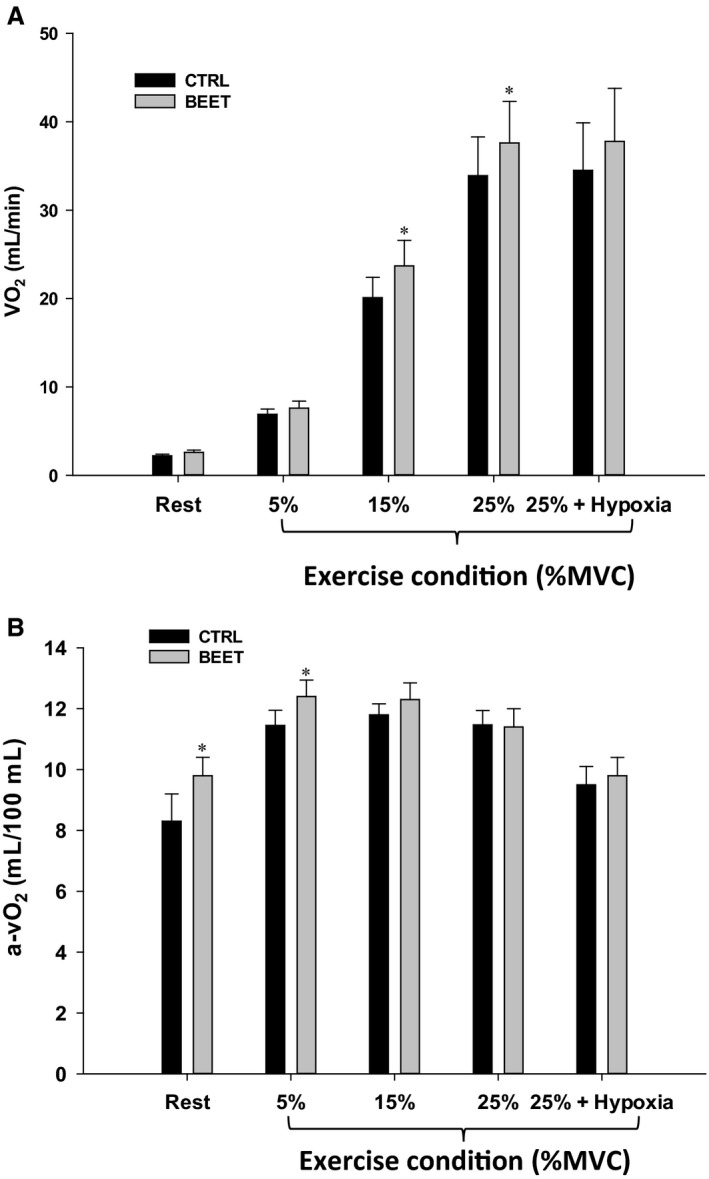
(A) Forearm VO_2_ and (B) a‐vO_2_ at rest and during graded handgrip exercise and hypoxic exercise prior to and following BR ingestion (BEET). *P < 0.05 versus control trial. % MVC, % of maximal voluntary contraction.

VO_2_ was not different at rest or during 5% exercise (Table 2; Fig. 4A). VO_2_ was significantly greater in the BR condition at both 15% MVC (24 ± 3 vs. 20 ± 2 mL/min) and 25% MVC (38 ± 5 vs. 34 ± 4 mL/min, all *P* < 0.05; Table 2; Fig. 4A). At rest (9.8 ± 0.7 vs. 8.3 ± 0.9 mL/100 mL *P* < 0.05) and during 5% MVC (12.4 ± 0.5 vs. 11.4 ± 0.5 mL/100 mL blood), there was a significantly greater a‐vO_2_ following BR ingestion (Fig. 4B), however, limb VO_2_ was not different during these conditions. There were no significant differences in the a‐vO_2_ difference at 15% MVC, 25% MVC, or 25% MVC + Hypoxia in control or BR conditions (Fig. 4B). In the placebo trial there were no significant differences in forearm VO_2_ at any time point within the two conditions (Control vs. Placebo; Table 2).

## Discussion

The key finding from the present study is that acute dietary ${\text{NO}}_{3}^{-}$ supplementation via BR significantly elevates skeletal muscle BF during moderate‐ to high‐intensity handgrip exercise in normoxia and systemic hypoxia in young adults. This elevation in BF is due to local vasodilation, as this increase was associated with a greater FVC. A secondary observation was that dietary ${\text{NO}}_{3}^{-}$ did not reduce forearm VO_2_ during graded handgrip exercise. To the best of our knowledge, this is the first study to determine whether dietary ${\text{NO}}_{3}^{-}$ ingestion via BR affects both skeletal muscle blood flow and local tissue oxygen consumption during exercise in humans.

### Effects of BR on FBF and FVC during normoxic and hypoxic exercise conditions

Recently, Ferguson et al. (2013) demonstrated that 5 days of dietary ${\text{NO}}_{3}^{-}$ supplementation via BR significantly increased skeletal muscle blood flow during exercise in healthy rats, and this was due to an elevation in vascular conductance. Our present observations support these findings, and indicate that acute BR does not impact forearm hemodynamics at rest and during low‐intensity exercise, but increases muscle blood flow via vascular conductance during moderate‐ to high‐intensity handgrip exercise in normoxia and hypoxia. There are several observations that merit discussion. First, studies performed in the rat hind limb during treadmill exercise indicate that the effects of BR predominantly occurred in fast twitch muscle fibers (Ferguson et al. 2013), presumably due to a more hypoxic and acidic environment which could increase the activity of key reductase enzymes involved in converting ${\text{NO}}_{2}^{-}$ to NO (Crawford et al. 2006; Zweier et al. 1995). Although we cannot speak to muscle fiber type‐specific effects of BR, it is of interest that the improvements in FBF and FVC we observed only occurred during moderate‐to‐high intensity exercise (~40% and 70% WRmax) (Richards et al. 2014), where a greater percentage of fast twitch fibers would be recruited. Second, although we demonstrate a significant increase in FBF and FVC during exercise with BR, the magnitude of increase is modest (~12–15%) compared with that observed in the hind limb of rats (~35–50%). The reason(s) for such a large discrepancy are unclear, but may relate to the duration of ${\text{NO}}_{3}^{-}$ supplementation (5 days vs. acute [2–3 h]), the exercise modality (handgrip exercise vs. treadmill exercise), and/or muscle fiber‐type composition in the rat hind limb versus human forearm (Delp and Duan 1996; Ferguson et al. 2013; Johnson et al. 1973). Finally, our observations are consistent with early studies using direct intra‐arterial nitrite infusion during exercise and hypoxia in humans (Cosby et al. 2003; Maher et al. 2008).

To our knowledge, this is the first study to observe a significant increase in muscle BF and VC during exercise following BR ingestion in young healthy adults. Two previous studies failed to demonstrate an effect of BR on exercising muscle BF or VC in young adults, however, some differences in the experimental approaches exist that may explain the discrepant findings. First, the magnitude of increase in plasma ${\text{NO}}_{2}^{-}$ in the previous studies were 1.6‐fold (Kim et al. 2015) and approximately twofold (Casey et al. 2015), whereas the increase in the present study was approximately fourfold above control (pre‐BR) conditions. Second, the subjects in the study by Casey et al. (2015) performed a similar handgrip exercise, but they only performed exercise at 20% MVC for 5 min. In contrast, subjects in the present study performed handgrip exercise for 4 min each at 5%, 15%, and 25% MVC and for an additional 3 min at 25% during hypoxia, and thus the exercise was graded and the total time of exercise was 10 min greater. Thus, it is possible that the graded nature of the exercise protocol or exercise duration may have differentially impacted the effect of acute BR. Finally, the subjects in the study by Kim et al. (2015) performed rhythmic handgrip exercise at very low workloads, the highest workload being equivalent to our lowest workload where we observed no effect of BR on exercising muscle BF or VC. Future studies may be needed to address the effect of exercise duration and intensity on the ability of dietary ${\text{NO}}_{3}^{-}$ supplementation to increase muscle BF and VC in humans.

### Effects of BR on forearm VO_2_ during normoxic and hypoxic exercise conditions

Previous studies in humans have demonstrated that dietary ${\text{NO}}_{3}^{-}$ supplementation can reduce the metabolic cost of exercise and improve exercise tolerance. These studies have utilized walking, running, and cycling coupled with measures of pulmonary VO_2_ (Bailey et al. 2015; Jones 2014; Lansley et al. 2011a,b; Vanhatalo et al. 2010; Wylie et al. 2013a) and, on average, demonstrate that VO_2_ for a given workload is ~5% lower with BR (Jones 2014). To the best of our knowledge, no studies have determined the effect of dietary ${\text{NO}}_{3}^{-}$ on muscle blood flow and VO_2_ in the exercising limb simultaneously in animals or humans. Based on the data in humans, we hypothesized that VO_2_ during exercise would be reduced with BR. In contrast, we found that the improvement in muscle BF and O_2_ delivery was associated with an increase in VO_2_ during moderate‐ to high‐intensity exercise. However, there was also a trend for an increase in VO_2_ in the placebo group during the second exercise trial at 25% + Hypoxia exercise intensity (*P* = 0.08), and the magnitude was quite similar to the subjects who ingested BR (Table 2). Despite this, it is important to note that there was not a similar trend for increases in muscle BF and VC in the placebo group. Collectively, our findings indicate that acute ${\text{NO}}_{3}^{-}$ supplementation increases muscle BF and VC, but does not reduce local tissue VO_2_, during rhythmic handgrip exercise in healthy humans. This increase in muscle perfusion and O_2_ delivery could help explain improvements in exercise tolerance by improving metabolic control (Bailey et al. 2009; Glean et al. 2015; Kenjale et al. 2011; Larsen et al. 2010; Vanhatalo et al. 2011).

### Experimental considerations

There are several experimental considerations that deserve attention, and the first relates to the use of a small muscle mass to study the effects of dietary ${\text{NO}}_{3}^{-}$ on exercising muscle BF, VC, and VO_2_. Our findings regarding the effects of ${\text{NO}}_{3}^{-}$ on muscle BF and VC are consistent with recent findings in the rat hind limb, however, in this study the investigators did not measure VO_2_ (Ferguson et al. 2013). Our data regarding muscle VO_2_ during handgrip exercise contrasts with what has been demonstrated with pulmonary measures of O_2_ uptake during larger muscle mass exercise (e.g., cycling, running, walking) (Bailey et al. 2015; Jones 2014; Lansley et al. 2011a,b; Vanhatalo et al. 2010; Wylie et al. 2013a). Thus, the type of exercise and muscle mass engaged must be considered when integrating these findings. Given that the studies during larger muscle mass exercise have not determined muscle blood flow, it remains unknown whether improvements in exercise tolerance under these conditions is due, in part, to improved skeletal muscle perfusion and O_2_ delivery. Furthermore, it remains unclear whether the reduction in pulmonary VO_2_ under these conditions is due solely to changes at the level of the active skeletal muscle. Future studies will be needed to address these issues.

A second consideration relates to the lack of hypoxia in the present study to evoke a greater increase in FBF and FVC in the BR compared with control conditions (Fig. 3). As expected, hypoxia in combination with exercise evoked a greater vasodilation and BF response compared with normoxia (Casey et al. 2010; Crecelius et al. 2011; Dinenno 2016), but this was not augmented with ${\text{NO}}_{3}^{-}$ supplementation and there was a parallel increase similar to control conditions. Given that reductase enzyme activity is elevated in conditions of low O_2_ (Crawford et al. 2006; Lundberg et al. 2008; Zweier et al. 1995), we had hypothesized that muscle BF would be greater in the BR trial under hypoxic exercise conditions. The lack of a further increase in FBF during hypoxic exercise in the BR condition could be attributed to greater degree of O_2_ delivery occurring prior to eliciting systemic hypoxia. During 25% MVC exercise, FBF and thus O_2_ delivery was ~15% higher in the BR versus control conditions and therefore the metabolic perturbation elicited by systemic hypoxia may not have been equal between the two conditions.

Finally, within the literature, there are variable findings related to the beneficial effect of dietary ${\text{NO}}_{3}^{-}$ supplementation via BR. Some studies suggest that alterations in VO_2_ are exercise intensity dependent (Bailey et al. 2015; Betteridge et al. 2016; Breese et al. 2013), and others indicate a training effect such that BR has less of an impact in highly endurance trained individuals (Boorsma et al. 2014; Wilkerson et al. 2012). While it is presently unclear why some subjects are more likely to respond to BR than others (Boorsma et al. 2014), training status and/or baseline plasma ${\text{NO}}_{3}^{-}$ and ${\text{NO}}_{2}^{-}$ levels could play a role. Participants in the present study were recreationally active to moderately trained, and none were highly trained, as assessed by physical activity questionnaires. We did not observe a correlation between baseline ${\text{NO}}_{3}^{-}$ and ${\text{NO}}_{2}^{-}$ and the increases in FBF and FVC after BR ingestion. However, we did observe a significant correlation between the percent increase in plasma ${\text{NO}}_{3}^{-}$ and percent increase in FBF during 25% MVC exercise (*r* ^2 ^= 0.49; *P* < 0.05), but not between ${\text{NO}}_{2}^{-}$ and FBF (*r* ^2^ = 0.07; NS). With respect to variability, of the 11 participants that received BR, two subjects exhibited <5% change in FBF (absolute Δ: 2–12 mL/min), five exhibited a 5–20% increase (absolute Δ: 14–24 mL/min), and four exhibited a >20% increase (absolute Δ: 64–75 mL/min). Thus, our study also highlights the variable effects of dietary ${\text{NO}}_{3}^{-}$ among individuals, and with respect to the present findings, another key issue to consider is the muscle fiber‐type distribution given the effects of dietary ${\text{NO}}_{3}^{-}$ may be more selective to fast twitch fibers (Ferguson et al. 2013, 2015).

### Potential implications

Although the present findings support the positive effect of dietary ${\text{NO}}_{3}^{-}$ on exercising muscle blood flow in young healthy adults, there is growing interest in ${\text{NO}}_{3}^{-}$ supplementation or ${\text{NO}}_{2}^{-}$ therapy to improve exercise tolerance/performance and vascular function in older adults and various clinical populations such as heart failure, peripheral artery disease, chronic obstructive pulmonary disease, diabetes, and patients with angina (Boden et al. 2012; Glean et al. 2015; Kerley et al. 2015; Shepherd et al. 2015a,b; Zamani et al. 2015). Specific to limb blood flow and BR ingestion, Casey et al. observed improvements in hypoxic exercise vasodilation in older adults, thus eliminating the age‐associated impairment that occurred prior to BR ingestion (Casey et al. 2015). Additional studies have reported reductions in total peripheral resistance (Lee et al. 2015), reduced arterial stiffness (Kim et al. 2015), and improved flow‐mediated vasodilation (Lee et al. 2015) following BR ingestion. In general, there are a number of studies in humans and animals that report positive effects of dietary ${\text{NO}}_{3}^{-}$ supplementation on exercise tolerance and vascular function and continue to represent an exciting area of potential intervention.

## Conclusions

Acute dietary ${\text{NO}}_{3}^{-}$ supplementation via BR increases muscle BF during moderate‐ to high‐intensity rhythmic handgrip exercise in normoxia and hypoxia, and this is due to local vasodilation (i.e., increases in vascular conductance). Furthermore, this is not associated with a reduction in exercising muscle VO_2_. Given that this is the first study to measure both exercising limb hemodynamics and VO_2_, future studies are needed to determine whether these observations occur during larger muscle mass exercise given previous observations of lower pulmonary VO_2_ during exercise, suggestive of a reduced metabolic cost of exercising muscle. Additionally, the potential benefit of dietary ${\text{NO}}_{3}^{-}$ supplementation to improve skeletal muscle perfusion in disease states such as heart failure, hypertension, and peripheral artery disease in humans warrants further study.

## Conflict of Interest

None declared.
